# Risk factors for intubation and mortality in patients treated with high flow nasal cannula due to COVID-19 infection. Survival Analysis Study in a Northern Mexican Population

**DOI:** 10.1371/journal.pone.0296931

**Published:** 2024-03-15

**Authors:** José Antonio Luviano-García, Alejandro Loose-Esparza, Yodira Guadalupe Hernández-Ruíz, Miguel Ángel Sanz-Sánchez, Hector Jeovanny Maheda-García, Miguel Angel Sosa-Medellin, Arnulfo Garza-Silva, Maria Elena Romero-Ibarguengoitia

**Affiliations:** 1 Hospital & Critical Areas, Hospital Clínica Nova de Monterrey, San Nicolás de los Garza, Nuevo León, México; 2 Vicerrectoría de Ciencias de la Salud, Escuela de Medicina, Universidad de Monterrey, San Pedro Garza García, Nuevo León, México; 3 Research Department, Hospital Clínica Nova de Monterrey, San Nicolás de los Garza, Nuevo León, México; 4 General Management, Hospital Clínica Nova, San Nicolás de los Garza, Nuevo León, México; University of Palermo, ITALY

## Abstract

**Background:**

COVID-19-related acute hypoxic respiratory failure patients often use high-flow nasal cannula (HFNO) oxygen therapy. COVID-19 HFNO intubation and mortality risk factors are understudied in the Mexican population, so the aim was to study them.

**Methods:**

This retrospective study searched electronic medical records from March 2020 to June 2022 for patients with COVID-19 who required hospitalization and HFNO. Descriptive statistics, a survival curve analysis, and Cox proportional hazard models were used to determine predictor factors for intubation and mortality in patients with HFNO and COVID-19, respectively.

**Results:**

A total of 134 patients received HFNO treatment. Ninety-one (67.9%) were men with a mean (SD) age of 54.5 (17.9) years. Common medical history included obesity (n = 89, 66.4%) with a Body Mass Index (BMI) mean (SD) of 31.8 (5.9), hypertension (n = 67, 50.0%), type 2 diabetes (n = 55, 41.0%), and dyslipidemias (n = 43, 32.1%). The variables associated with a greater risk of requiring intubation after high-flow therapy were age (HR = 1.018, 95% CI 1.003–1.034, p = 0.022) and BMI (HR = 1.071, 95% CI 1.024–1.120, p = 0.003). No variables were associated with lower risk. Increased mortality was associated with increasing age (HR = 1.151, 95% CI 1.102–1.201, p = <0.001), hypertension (HR = 4.092, 95% CI 1.369–12.236, p = 0.012), and dyslipidemia (HR = 3.954, 95% CI 1.395–11.209, p = 0.010). Patients with type 2 diabetes had a lower risk of mortality (HR = 0.235, 95% CI 0.080–0.688, p = 0.008).

**Conclusions:**

A higher age and BMI were associated with an increased risk of intubation in patients with HFNO and COVID-19. Hypertension and dyslipidemias were associated with a higher risk of mortality.

## Introduction

The SARS-CoV-2 infected millions of subjects worldwide. The clinical presentation may vary from mild to severe. The World Health Organization (WHO) advised regular monitoring of oxygenation in severe patients to determine the need for oxygen therapy [1]. About fourteen percent of individuals develop hypoxemic respiratory failure and require oxygen therapy, while five percent require even more intensive breathing assistance [2]. The spread of the virus exceeded hospitals’ capacity for moderate and severe patients by mid-2020, resulting in a global shortage of mechanical ventilators. Since most COVID-19 patients died from severe hypoxemia, testing new low and high-flow ventilation therapies was necessary to provide essential respiratory support [3].

High-flow nasal cannula oxygen (HFNO) is a non-invasive therapy to support patients experiencing severe and life-threatening hypoxemic respiratory failure. Its primary purpose is to generate flows of up to 60 l/min, allowing for fractional-inspired oxygen concentrations of up to 90%. Improvements in oxygenation, the metabolic requirement for respiration, carbon dioxide production, comfort, the effort of breathing, and positive nasopharyngeal and tracheal airway pressure, are just a few of the physiological benefits of HFNO [4–8]. Critical patients can be handled with high-flow ventilation; otherwise, mechanical ventilation is needed [9].

Multiple studies have reported the benefits of HFNO in reducing the need for mechanical ventilation, as evidenced by lower intubation rates [2, 10, 11]. Leroux et al. reported in their study that in 47.8% of cases, HFNO prevented the need for intubation [2]. Additional studies have found that HFNO may enhance the prognosis and decrease mortality [2, 12].

There is limited published literature on predicting outcomes such as intubation and mortality in subjects with COVID-19 infection treated with HFNO in the Mexican population. This population has a high prevalence of obesity, diabetes, and hypertension, which could compromise clinical behavior in respiratory failure [13]. Therefore, the study aimed to identify patients’ risk factors for intubation and mortality in Mexican individuals using HFNO.

## Materials and methods

### Study population and design

From March 2020 to June 2022, patients diagnosed with COVID-19 were hospitalized at Hospital Clinica Nova (HCN), a private hospital in Northeastern Mexico. The population of HCN consists of employees from enterprises related to the Ternium group, their families, and retired employees. With a population at high risk for COVID-19, the hospital performed a retrospective observational study that included patients with COVID-19 who required high-flow ventilation while hospitalized. The study followed the STROBE guidelines and received approval from the local Institutional Review Board (17102022-CN-NEUM-CI) [14]. Medical charts were reviewed and analyzed from November 2022 –May 2023. The use of a consent form did not apply due to the retrospective nature of this study.

The inclusion criteria were hospitalized patients of both genders, adults (>18 years), and confirmed COVID-19 infection by real-time reverse transcription-polymerase chain reaction (RT-PCR) or rapid antigen test. Patients who received HFNO for less than 24 hours were excluded from the study. Also, patients who died within the first 24 hours were excluded.

All patients underwent a standardized protocol based on peripheral oxygen saturation levels. If the saturation was between 90% and 94%, nasal prongs were administered with a maximum flow of 10 lpm. If oxygen saturation dropped below 90%, patients were transitioned to an oxygen mask. If it became difficult to maintain oxygen saturation above 90%, they were subsequently provided with a reservoir mask, and if saturation still couldn’t be maintained above 90%, HFNO was initiated. Subjects exhibiting tachypnea (> 30 bpm) and oxygen saturation levels between 80% and 90% could be directly treated with HFNO. If their tachypnea was greater than 30 bpm, their oxygen saturation fell below 80%, or the Rox index was less than 3, intubation was performed.

The VAPOTHERM PRECISION FLOW (USA) system was used for HFNO. High-flow ventilation began with a flow of 20 L/min and FiO2 at 1.0, with both flow and FiO2 adjusted to maintain a peripheral saturation of 90–94% and adjusted dynamically.

Data such as age, sex, body mass index (BMI), previous diagnoses of diabetes mellitus, systemic arterial hypertension, and heart disease, among others, were analyzed from the medical records. The number of days of the evolution of COVID-19 infection before admission, the type of oxygen supplementation at admission and through hospitalization, their hospital stays, and the number of days between the oxygen supplementation and death or discharge to analyze their clinical outcome were also obtained.

During the hospital stay, viral load, complete blood count, lactate dehydrogenase (LDH), interleukin-6 (IL-6), C-reactive protein (CRP), D-dimer, and ferritin were taken on the admission day and constantly every 24–48 hours. The nursing personnel took the blood sample through a peripheral venous puncture.

### Statistics analysis

The distribution of variables was evaluated with the Shapiro-Wilk test or Kolmogorov test; the necessary transformation for their normalization was performed. The descriptive analysis of the variables was performed using frequencies, percentages, mean and standard deviations (SD), if they conformed to normality, or median and interquartile range (IQR) otherwise. Survival curve analysis and Cox proportional hazard models were used to determine predictive factors for intubation and mortality in patients with HFNO associated with COVID-19. Missing values were managed through complete case analysis. A value of p<0.05 indicates statistical significance. Data were analyzed with SPSS version 22.

## Results

From March 2020 to June 2022, 650 COVID-19 patients were hospitalized, with 146 receiving HFNO treatment. After excluding 4 patients who died within 24 hours of admission and 8 who used HFNO for less than 24 hours, the study focused on 134 patients who exclusively received HFNO for more than 24 hours.

Of the patients studied, 67.9% (n = 91) were men with a mean (SD) age of 54.5 (17.9) years. Among the medical history, the most prevalent diseases were obesity (n = 89, 66.4%), systemic arterial hypertension (n = 67, 50.0%), diabetes mellitus type 2 (n = 55, 41.0%), and dyslipidemias (n = 43, 32.1%). Concerning the COVID-19 vaccination schedule, only 16 (11.9%) patients reported being immunized. Most received CoronaVac (n = 8, 6.0%) and ChAdOx1-S (AstraZeneca) (n = 6, 4.5%) as the first dose. Table 1 describes the demographic data on hospitalized patients with COVID-19.

**Table 1:** Demographic data of hospitalized patients with COVID-19.

n = 134	Frequencies (%)
Age	54.5 (17.9)^a^
**Gender**	
Female	43(32.1)
Male	91(67.9)
**Medical History**	
Weight	87.5(17.9)^a^
Height	1.6(0.1)^a^
Body Mass Index (IMC)	31.8 (5.9)^a^
Normal	13(9.7)
Overweight	31(23.1)
Obese	89(66.4)
Obese Class I	58(43.3)
Obese Class II	20(14.9)
Obese Class III	11(8.2)
Hypertension	67(50.0)
Diabetes Mellitus Type 2	55(41.0)
Prediabetes	24(17.9)
Smoking	16(11.9)
Heart diseases	9(6.7)
Hypothyroidism	10(7.5)
Asthma	4(3.0)
Dyslipidemias	43(32.1)
Active neoplasm	5(3.7)
Previous neoplasm	5(3.7)
**SARS-CoV-2 vaccination schedule**	
Vaccinated patients	16(11.9)
First dose	
Pfizer	1(0.7)
AstraZeneca	6(4.5)
Johnson & Johnson	1(0.7)
Coronavac	8(6.0)
Second dose	
Pfizer	1(0.7)
AstraZeneca	4(3.0)
Johnson & Johnson	0(0.0)
Coronavac	3(2.2)

Data presented in frequencies and percentages.

^a^Shown in mean and standard deviation (SD).

COVID-19 infection developed over a median (IQR) of 9.0(3.0) days before admission. Patients’ hospital stay had a median (IQR) of 13.0(13.0) days. On the first day of treatment, patients receiving high flow had an SPO_2_ median (IQR) of 95.0(4.0)%, and an FIO_2_ median (IQR) of 65.0(40.0)%. The median (IQR) duration for high-flow therapy patients was 3.0(3.0) days. Meanwhile, the median (IQR) days between high flow and intubation were 10.6(5.7). The median (IQR) days between intubation and death for intubated patients was 6.2(3.1). Laboratory reports showed a CPR mean (SD) of 9.74 mg/L (1.3), ferritin mean (SD) levels of 1067.0 mg/ml (2.9), IL-6 of 47.0 pg/ml (6.8), D-dimer of 722.7 ng/ml (2.2), and LDH of 429.5 UI/L (178.8), and lymphocytes of 745.2(3.4). Table 2 reports the laboratory data on hospitalized patients. Concerning glucose levels between subjects with type 2 diabetes and the ones without this condition, on the first day of hospitalization, the mean was 169 (10) vs. 121(10) mg/dl (p< 0.001). On the day of discharge from the hospital, it was 129 (10) vs. 113mg/dl (p = 0.075), and the A1c was 7.5% vs. 6.0% (p = 0.01).

**Table 2:** Laboratory data of hospitalized patients with COVID-19.

n = 134	Mean (SD)
C-reactive protein on the first day with high flow, mg/L	9.74(1.3)
Ferritin on the first day with high flow, mg/ml	1067.0(2.9)
IL-6 on the first day with high flow, pg/ml	47.0(6.8)
D-Dimer on the first day with high flow, ng/ml	722.7(2.2)
Lactate dehydrogenase on the first day with high flow, UI/L	429.5(178.8)
Lymphocytes on the first day with high flow	745.2(3.4)
Lymphocytes by the end of the hospitalization	1096.4(2.6)
Initial viral load copies/ml	2195.3(7.3)
Final viral load copies/ml	148.3(5.4)

Data presented in means and standard deviations.

We analyzed COVID-19 severity through the Total Severity Score (TSS), a CT evaluation to assess inflammatory abnormalities like mixed ground-glass opacities, ground-glass opacities, and consolidations in all five lobes of both lungs. This score assigns a value between 0 and 4 to each lobe based on the extent of involvement: 0 signifies no involvement, 1 indicates 1% to 25% involvement, 2 represents 26% to 50% involvement, 3 signifies 51% to 75% involvement, and 4 represents 76% to 100% involvement. The TSS is calculated by adding the individual lobe scores, resulting in a score ranging from 0 to 20 points [15]. Our study showed no statistical difference in the TSS between patients who were intubated and those who did not (p = 0.194), as well as between subjects who died and those who did not (p = 0.116).

Additionally, we analyzed the ROX index, which calculates the ratio of oxygen saturation measured by pulse oximetry to the oxygen fraction (FiO2) divided by the respiratory rate. Given its validation in patients experiencing acute hypoxemic failure, it is regarded as a valuable asset in the context of HFNC treatment [16]. We measured the ROX index 12 hours after starting HFNO, revealing that individuals who didn’t survive had an average value of 4.3 (1.5), whereas those who survived had an average of 5.4 (1.5), with a statistically significant difference (p = 0.007).

**Fig 1:**
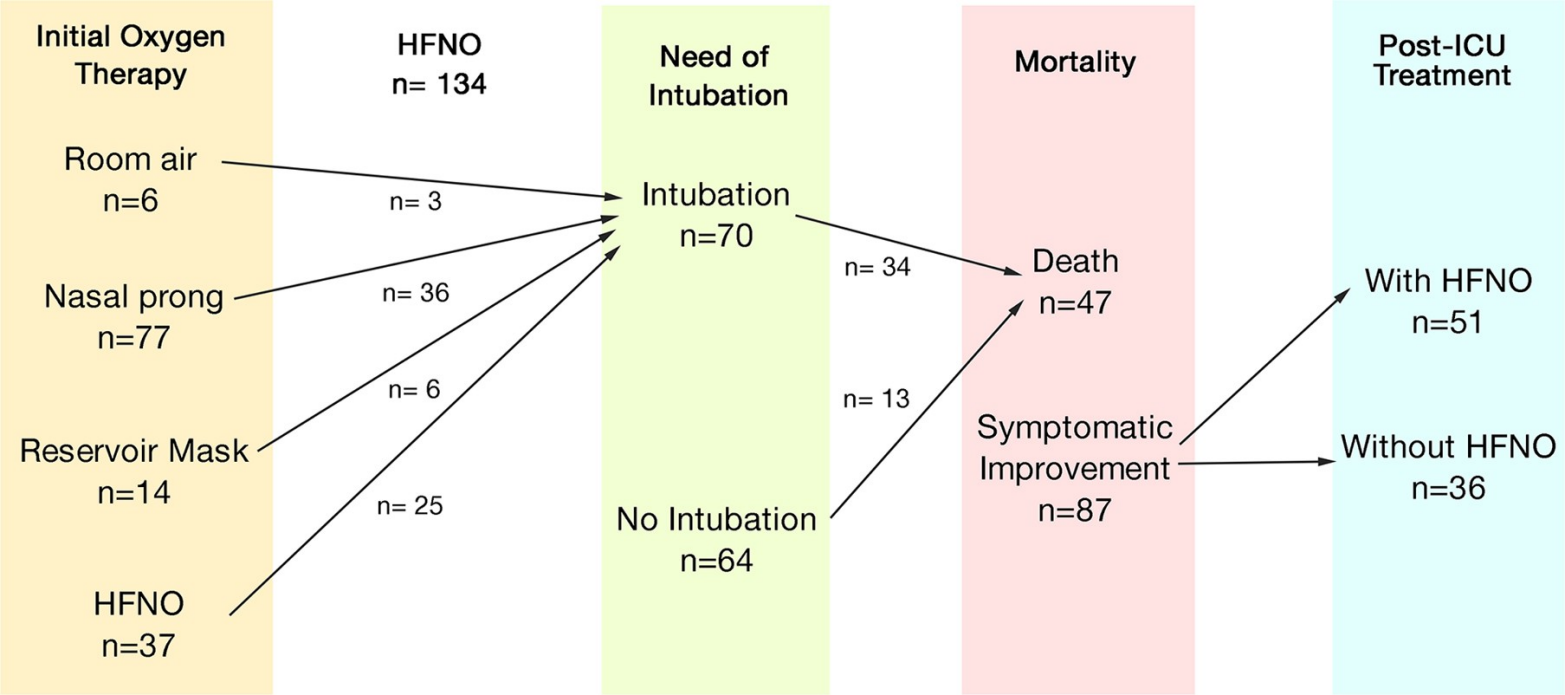
Clinical evolution of HFNO-treated subjects.

Initial management for hospitalized COVID-19 patients could consist of room air (n = 6, 4%), nasal prong (n = 77, 57%), reservoir mask (n = 14, 10%), or HFNO (n = 37, 27%). Regarding the outcomes, 70 (52.2%) patients required intubation during hospitalization. Forty-seven (35%) patients died during the follow-up, from which 34 (25%) were on mechanical ventilation. Of the discharged patients, 51 (38.1%) were sent home with high-flow therapy. Fig 1 shows the clinical evolution of HFNO-treated subjects.

### Survival Analysis and Cox proportional hazard model

A total of 134 individuals were included in the adjusted COX proportional hazard model; from which 77 (57.5%) patients required nasal prong as initial oxygen therapy, 37 (27.6%) initially required high-flow therapy, and 70 (52.2%) were intubated. The variables associated with a greater risk of requiring intubation after high-flow therapy were older age (HR = 1.018, 95% CI 1.003–1.034, p = 0.022) and higher BMI (HR = 1.071, 95% CI 1.024–1.120, p = 0.003) There were no variables associated with lower risk. Table 3 describes the adjusted Cox proportional hazard model for the risk factors of requiring intubation after high-flow therapy.

**Table 3:** Adjusted Cox proportional hazard model for risk factors for intubation.

	B	Standard error	*p*-value	HR	95% CI
**Age**	0.018	0.008	0.022	1.018	1.003–1.034
**Gender**	-0.139	0.276	0.615	0.870	0.507–1.496
**Body Mass Index**	0.068	0.023	0.003	1.071	1.024–1.120
**Total severity score**	0.222	0.170	0.191	1.248	0.895–1.741

Dependent variable: Intubation

HR: Hazard ratio.

CI = confidence interval.

On the other hand, the variable associated with a lower risk of death after being hospitalized is the presence of diabetes mellitus type 2 (HR = 0.235, 95% CI 0.080–0.688, p = 0.008). The variables associated with a greater risk of death after being hospitalized are older age (HR = 1.151, 95% CI 1.102–1.201, p = <0.001), the presence of hypertension (HR = 4.092, 95% CI 1.369–12.236, p = 0.012) and dyslipidemia (HR = 3.954, 95% CI 1.395–11.209, p = 0.010). Rox index and TSS were initially considered in the models; however, we found no effect, so they were removed from the final model. Table 4 describes the adjusted Cox proportional hazard model for the risk factors of death after being hospitalized.

**Table 4:** Adjusted Cox proportional hazard model for risk factors for mortality.

	B	Standard error	*p*-value*	HR	95% CI
**Age**	0.140	0.022	<0.001	1.151	1.102–1.201
**Gender**	0.193	0.439	0.660	1.213	0.513–2.868
**Hypertension**	1.409	0.559	**0.012**	4.092	1.369–12.236
**Diabetes Mellitus**	-1.450	0.549	0.008	0.235	0.080–0.688
**Dyslipidemia**	1.375	0.532	0.010	3.954	1.395–11.209
**C-reactive protein**	-0.608	0.412	0.140	0.545	0.243–1.221
**Lactate dehydrogenase**	0.001	0.001	0.259	1.001	0.999–1.004
**IL-6**	0.160	0.319	0.615	1.174	0.628–2.193

Dependent variable: Mortality.

HR: Hazard ratio.

CI = Confidence Interval.

## Discussion

This study evaluated the risk factors for intubation and mortality in Mexican individuals with COVID-19 who received HFNO. The results revealed age and BMI were significant risk factors for requiring intubation after high-flow therapy. Gender, total severity score, and intubation risk were unrelated. Age, hypertension, and dyslipidemia increased hospitalization-related mortality in elderly patients. Diabetes mellitus type 2 had a protective effect against mortality.

Age appears to be a substantial risk factor for severe COVID-19 outcomes. The results showed that the likelihood of needing intubation increased by 1.8% per year of age. Multiple international studies demonstrated an increased probability of severe outcomes with age [17–19]. A Mexican study by Fernando Mesta et al. demonstrated an increased probability of severe outcomes with age [20]. Due to age-related declines in immunological function and an increased prevalence of comorbidities, the elderly may be at increased risk for developing life-threatening illnesses [17]. Individuals over the age of 65 are more likely to have underlying health problems, diminished physiological reserve, and weakened immune function, making them more prone to severe disease and its repercussions [18].

BMI was another significant risk factor for intubation in the investigation. Each unit increase in BMI was correlated with a 7.1% increase in relative risk. These results are consistent with the accumulating body of evidence that identifies obesity as a risk factor for COVID-19 outcomes with severe consequences. Obesity is associated with chronic inflammation, impaired immune function, and increased rates of comorbidities, including diabetes, cardiovascular disease, and respiratory conditions. Several international studies, including those by Simonnet et al. and Popkin et al., have identified obesity as a significant risk factor for severe COVID-19 outcomes, such as the requirement for invasive mechanical ventilation [21, 22].

Intriguingly, it was found that type 2 diabetes was protective against mortality in COVID-19 hospitalized patients. This result contradicts the findings of other studies that identified diabetes as a risk factor for serious outcomes [23–26]. However, it is important to note that diabetes is a complex disease with distinct subtypes, and its impact on COVID-19 outcomes varies.

Within our cohort, it is noteworthy that despite subjects with diabetes displaying slightly higher statistically significant mean glucose levels on admission than those without diabetes, the mean did not exceed 180mg/dl, and the mean A1c was 7.5%. Upon discharge, there was no statistical difference in the mean glucose levels between both groups, which remained below 180mg/dl. This suggests that the majority of subjects with Type 2 diabetes had moderate glucose control before admission and achieved very good control during their hospitalization. Interestingly, subjects without diabetes had mean glucose levels on admission that exceeded 100mg/dl, possibly attributable to the stress associated with COVID-19 infection.

A possible explanation for the relative protection of the patients with diabetes in this study could implicate the different effects of stress-induced hyperglycemia between the two groups. Hyperglycaemia is common in critically ill patients and not only in those with diabetes. It is associated with mortality, but it has been shown that patients with diabetes are less affected than nondiabetic patients by high glucose levels. It could be that the relative protection from stress-induced hyperglycemia counteracts the increased mortality risk due to an increased amount of complications [27–30].

During the research process, data showed that hypertension and dyslipidemia were additional risk factors for mortality. High blood pressure is the most prevalent risk factor in Mexico. At least ⅓ of the adults over 20 years old have this condition and it is highly linked to cardiovascular death in our country [31]. Multiple factors have been related to hypertension; however, 30–40% of the variation in blood pressure is also related to genetics [32]. Additionally, a previous meta-analysis showed a higher proportion of hypertension in critically ill subjects with COVID-19 infection vs. non-critical and a high association with death (2.17-fold higher risk). Other studies have confirmed the same association [33, 34].

Researchers have suggested that subjects with hypertension may experience a decreased ACE2 expression, which, when bound by SARS-CoV-2, attenuates residual ACE2, leading to elevated angiotensin II levels, driving the development of COVID-19 and its severity [35]. Endothelial dysfunction, inflammation, and an increased risk of thrombotic events may be additional mechanisms. Both Guan et al. and Wang et al. conducted research that found comparable connections between high blood pressure, abnormal lipid levels, and poor COVID-19 outcomes [36, 37].

A previous meta-analysis showed an increased mortality risk (2.13-fold) in subjects with dyslipidemia who got infected with COVID-19 [38]. Hypercholesterolemia is an independent risk for cardiovascular mortality and inflammation [39]. Low-density lipoprotein (LDL) forms oxidized LDL (oxLDL) after crossing the endothelial barrier with increased oxidative stress. OxLDL can form immune complexes and can activate endothelial cells and monocytes, increasing the expression of a variety of inflammatory proteins and receptors [40]. OxLDL can also induce endothelial cell apoptosis through LDL caspase-3, caspase-9, receptor-1-mediated NF-κB signaling, and Fas, thus increasing monocyte levels, platelet activation, and vascular smooth muscle cell migration induced by collagen exposure [41, 42]. Pathological results have shown that the lung injury of COVID-19 patients is caused by endothelial cell apoptosis [43]. Dyslipidemia promotes endothelial dysfunction and activation, which leads to the increase of pro-inflammatory cytokines and reactive oxygen species. Additionally, it is known that hyperlipidemia damages the immune response and may lead to persistent chronic inflammation, which leads to cardiovascular disease risk [44]. So, the combination of hypertension, dyslipidemia, and COVID-19 infection generates an additive effect that increased mortality in our study group through endothelial damage and inflammation.

Certain biochemical markers were discovered as predictors of mortality in a study of intubated COVID-19 patients in Mexico. The non-survivor group had greater levels of lactate dehydrogenase (LDH) and D-dimer, according to the study [45]. Furthermore, research on hospitalized COVID-19 patients conducted in Mexico by Arturo Cortés-Tellés et al. revealed significant risk factors for mortality. A neutrophil-to-lymphocyte ratio (NLR) ≥9, low albumin levels, high LDH levels, and the necessity for invasive mechanical ventilation (IMV) were among these causes [46]. Correlation between inflammatory markers and worse outcomes, such as intubation or mortality, was not found in the regression models.

Concerning the TSS index, even though a previous meta-analysis has shown that several tomographic scores help correlate mortality in COVID-19, the specific scale we used did not find any relation [47]. We will have to re-evaluate a new score that can correlate with this outcome in the future in our population. Approximately 12 hours after measuring the ROX index with HFNO, the t-test analysis revealed that individuals who didn’t survive had a value below the established cut-off point of 4.88. This value, as mentioned in the literature, is associated with HFNO failure [48]. However, the Cox-regression model did not associate this index with mortality.

### Limitations and future research

This study had some limitations worth mentioning. It was a retrospective investigation and clinicians established beforehand how to administer the HFNO. The clinicians in attendance determined the transition between oxygen therapies. Diverse physicians have divergent opinions regarding the transition to intubation. However, this investigation can show how the HFNO has been utilized among COVID-19 patients in the real world and the risk factors for worse outcomes such as intubation and mortality could be evaluated. All COVID-19 patients who met the profile to fit in the study during the investigators’ period in the hospital were included; however future confirmatory studies with an increased sample size are required. It’s expected to provide an accurate picture of HFNO treatment for COVID-19 for future reference when using HFNO to treat COVID-19 patients with hypoxic respiratory failure.

## Conclusion

A higher age and BMI were associated with an increased risk of intubation in patients with HFNO and COVID-19. In this cohort of northern Mexican subjects, hypertension, and dyslipidemia were associated with a higher risk of mortality.

## Supporting information

S1 Data (XLSX)

## List of abbreviations

WHO: World Health Organization
HFNO: High-flow nasal cannula oxygen
HCN: Hospital Clinica Nova
RT-PCR: Real-time reverse transcription-polymerase chain reaction
BMI: Body mass index
LDH: Lactate dehydrogenase
IL-6: Interleukin-6
C-RP: C-reactive protein
SD: Standard deviations
IQR: Interquartile range
LDH: Lactate dehydrogenase
TSS: Total Severity Score
BPM: Breath per minute
